# Machine learning in the prediction of cancer therapy

**DOI:** 10.1016/j.csbj.2021.07.003

**Published:** 2021-07-08

**Authors:** Raihan Rafique, S.M. Riazul Islam, Julhash U. Kazi

**Affiliations:** a Ideflod AB, Lund, Sweden; b Department of Computer Science and Engineering, Sejong University, Seoul, South Korea; c Division of Translational Cancer Research, Department of Laboratory Medicine, Lund University, Lund, Sweden; d Lund Stem Cell Center, Department of Laboratory Medicine, Lund University, Lund, Sweden

**Keywords:** Artificial intelligence, Deep learning, Monotherapy prediction, Drug combinations, Drug synergy, Variational autoencoder, Restricted Boltzmann machine, Support vector machines, Ridge regression, Elastic net, Lasso, Random forests, Deep neural network, Convolutional neural network, Graph convolutional network, Matrix factorization, Factorization machine, Higher-order factorization machines, Visible neural network, Ordinary differential equation

## Abstract

Resistance to therapy remains a major cause of cancer treatment failures, resulting in many cancer-related deaths. Resistance can occur at any time during the treatment, even at the beginning. The current treatment plan is dependent mainly on cancer subtypes and the presence of genetic mutations. Evidently, the presence of a genetic mutation does not always predict the therapeutic response and can vary for different cancer subtypes. Therefore, there is an unmet need for predictive models to match a cancer patient with a specific drug or drug combination. Recent advancements in predictive models using artificial intelligence have shown great promise in preclinical settings. However, despite massive improvements in computational power, building clinically useable models remains challenging due to a lack of clinically meaningful pharmacogenomic data. In this review, we provide an overview of recent advancements in therapeutic response prediction using machine learning, which is the most widely used branch of artificial intelligence. We describe the basics of machine learning algorithms, illustrate their use, and highlight the current challenges in therapy response prediction for clinical practice.

## Graphical abstract

**Figure:**
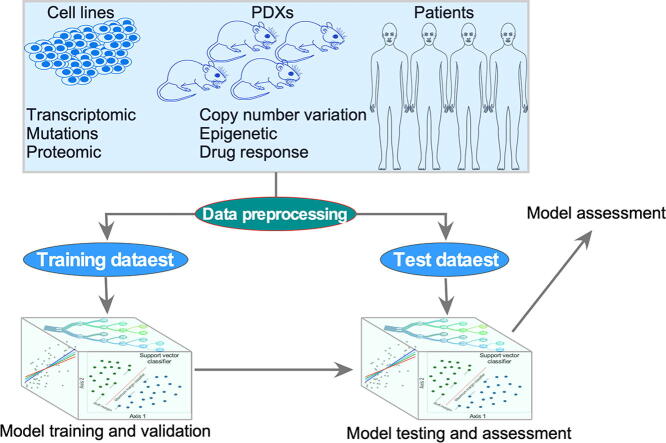

## 1 Introduction

Adaptive resistance mechanisms are highly dependent on cancer subtypes and applied treatments. Therefore, the resistance mechanism needs to be defined for each cancer subtype and individual treatment plan. Currently, hardly any tools exist to determine from the beginning whether a patient will respond to a specific therapy or display resistance. Thus, there is an unmet need to develop tools to identify drug responses in individual patients for precision medicine. Recent technological advances have initiated a new era of precision medicine through data-driven assessment of diseases by combining machine learning (ML) and biomedical science. The use of artificial intelligence such as ML helps to extract meaningful conclusions by exploiting big data, thereby improving treatment outcomes. ML is widely used in cancer research and is becoming increasingly popular for cancer detection and treatment. The main goal of precision medicine is to provide therapies that not only increase the survival chances of patients but also improve their quality of life by reducing unwanted side effects. This can be achieved by matching patients with appropriate therapies or therapeutic combinations.

Some of the early studies on ML and its applications in human cancer research have been discussed elsewhere [1]. Several recent overviews in this emerging field have provided valuable insights into the relevant computational challenges and advancements [2], [3], [4], [5], [6], [7], [8]. These overviews illustrated the importance of the field and supported the notion that ML is a highly promising approach to personalized therapy for cancer treatment. In a recent review, a broad perspective was provided on how ML tools can be incorporated into clinical practice with a focus on biomarker development [9]. Another review identified several challenges in omics data analysis and data integration to obtain robust results in big-data-assisted precision medicine [10]. Several other reviews dealt primarily with the computational methods and software that are required to advance data-driven precision oncology [11], [12], [13]. Also, whereas Grothen et. al. discussed artificial intelligence-based investigations into cancer subtypes and disease prognosis from a system biology perspective [14], Biswas et. al. reviewed artificial intelligence applications for pharmacy informatics in a surveillance and epidemiological context [15]. Another study systematically explained how deep learning (DL), a subset of ML, has emerged as a promising technique, highlighting various genomics and pharmacogenomics data resources [16]. However, the aforementioned studies did not focus strictly on drug response prediction from clinical perspectives. In recent years, several surveys and review articles have presented the potential and challenges of ML adoption in clinical practice and drug response prediction in cancer treatment [17], [18], [19], [20], [21], [22], [23]. Nonetheless, the area of applications of ML in cancer treatment is so diverse that various issues still need to be analyzed from a holistic perspective. In this review, we provide a comprehensive overview of the ML solutions for drug response prediction relating to the relevant clinical practices. In addition to discussing the basics of therapy response prediction and related ML principles, we systematically present the ML and DL approaches that are promising for monotherapy and combination therapy in cancer treatment, a focus that makes our article different from existing surveys and reviews.

## 2 Basics of therapy response prediction

**Fig. 1:**
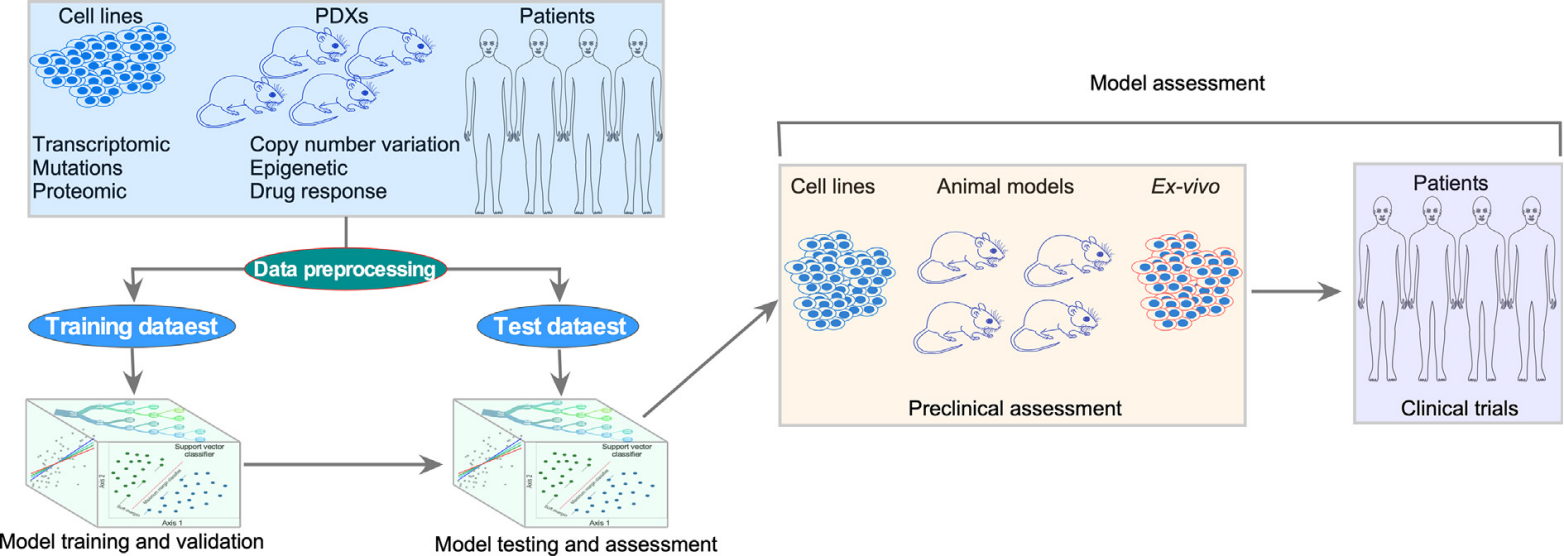
Workflow for ML prediction model development. Pharmacogenomic data from cell lines, patient-derived xenografts (PDXs), and patient materials are ideal for ML model development. Data from different sources are preprocessed and then divided into training (including cross-validation) and test groups. The training dataset is used to build and validate the prediction model, while the test dataset is used for testing the model’s accuracy and precision. To develop a prediction model for clinical use, vigorous preclinical assessment is required that can be performed using cell lines, PDXs, and patient materials that have not been used for model development. Additionally, the efficacy of predicted drugs must be tested for disease-specific preclinical models. Finally, both the model and predicted drug will undergo a clinical trial.

Predictive model development involves several steps that combine biological data and ML algorithms. A brief workflow has been depicted in Fig. 1.

### 2.1 Pharmacogenomic data resources

High-quality biological data are a prerequisite for a good model. Large-scale cell line data are publicly available from different platforms and include genomic, transcriptomic, and drug response data. Pharmacogenomic data for cell lines are available mainly from the Cancer Cell Line Encyclopedia (CCLE) [24], [25], NCI-60 [26], the Genomics of Drug Sensitivity in Cancer (GDSC) [27], [28], gCSI [29], and the Cancer Therapeutics Response Portal (CTRP) [30], [31]. PharmacoDB [32] and CellMinerCDB [33], [34] provide access to the curated data from different studies. These datasets offer baseline genomic and transcriptomic data for cell lines covering a wide range of cancers. DrugComb [35] and DrugCombDB [36] offer manually curated drug combination data from different studies. Besides these pharmacogenomic data for cell lines, which have been widely used to develop ML models, several initiatives have recently been undertaken to generate pharmacogenomic data from patient-derived xenografts (PDXs). Compared with cell lines, PDXs are superior in predicting clinical activities. PDX finder [37], PRoXE [38], PDMR [39], and EorOPDXs [40] provide comprehensive data for PDXs. Several other studies also provide high-quality transcriptomic and pharmacogenomic data that are useful for model development or testing when combined with other datasets [41], [42], [43], [44], [45].

### 2.2 Data preprocessing

Data preprocessing is an important step in the ML approach. Large-scale data preprocessing includes data selection, noise filtering, imputation of missing values, feature selection, and normalization.

*Data selection* – Data selection remains the most challenging aspect due to the possible inconsistencies between different datasets [46]. Studies comparing the largest public collections of pharmacological and genomic data for cell lines suggest that each dataset separately exhibits reasonable predictive power but that combining datasets can further increase the classification accuracy [29], [47].

*Feature selection* – Large-scale cell line datasets comprise transcriptomic, mutational, copy number variation (CNV), methylation, and proteomic data. Although genetic features such as mutations, CNV, and promotor methylation have been shown to provide important therapeutic insights, these features seem to be limited to individual tumors [27]. Therefore, it has been suggested that transcriptomic features alone hold the most predictive power and that the addition of genetic features marginally improves performance of an ML model [48], [49], [50]. The feature-to-sample ratio plays an important role in controlling the variances, with a smaller ratio providing better prediction [51]. However, maintaining a proper feature-to-sample ratio is challenging for pharmacogenomic data. For example, transcriptomic data can have more than 15,000 features, while the number of samples in any pharmacogenomic study remains between 100 and 1000. Systematically reducing the number of features (also known as dimensionality reduction) by incorporating meaningful descriptions improves prediction accuracy by reducing overfitting [52], [53]. Several techniques can be used for feature selection, including minimum redundancy maximum relevance (mRMR), high-correlation filters, principal component analysis, and backward feature elimination [54], [55], [56], [57], [58], [59], [60], [61], [62].

*Data normalization* – Because the range of values of raw data varies widely, a normalization technique (also known as feature scaling) is applied to change the values of numeric columns in the dataset to obtain a common scale, so that the associated objective functions work properly. Different ways exist to perform feature scaling, including min–max normalization, rank-invariant set normalization, data standardization, cross-correlation, and scaling to unit length [63].

## 3 ML algorithms for drug response prediction

**Fig. 2:**
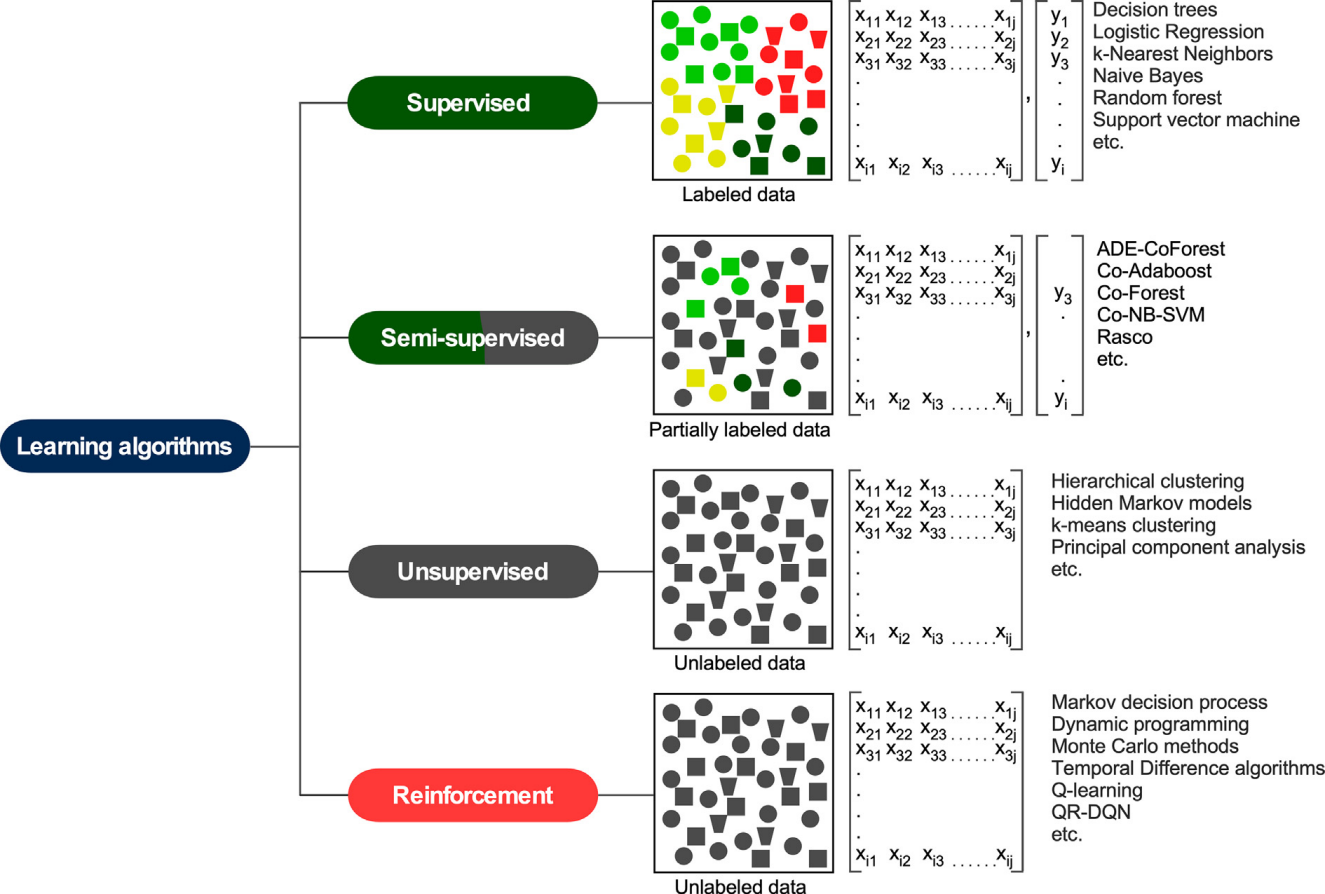
Schematic representation of different ML algorithms. In a supervised learning model, all data have a known label, while the semi-supervised model can handle partially labeled data. Both unsupervised and reinforcement learning algorithms can handle unlabeled data.

ML algorithms can be grouped into four major classes: supervised learning, semi-supervised learning, unsupervised learning, and reinforcement learning [64], [65]. Supervised learning algorithms use a training dataset with known outcomes to build a hypothetical function with decision variables that can later be used to predict unknown samples (Fig. 2). On the other hand, unsupervised learning algorithms use unlabeled data to find hidden structures or patterns; these algorithms are widely used in biological research for clustering and pattern detection. Semi-supervised learning algorithms are self-learning and can develop a prediction model from partially labeled data [66]. A reinforcement learning algorithm employs a sequential decision problem in which the algorithm solves a problem and learns from the solution [65]. In this case, the algorithm discovers which actions result in the best output on a trial-and-error basis. Perhaps supervised learning algorithms are generally used for building classification models, and these algorithms have also been widely tested for predicting treatment outcomes. Therefore, in this review, we will focus mainly on supervised learning algorithms.

### 3.1 Linear regression

**Fig. 3:**
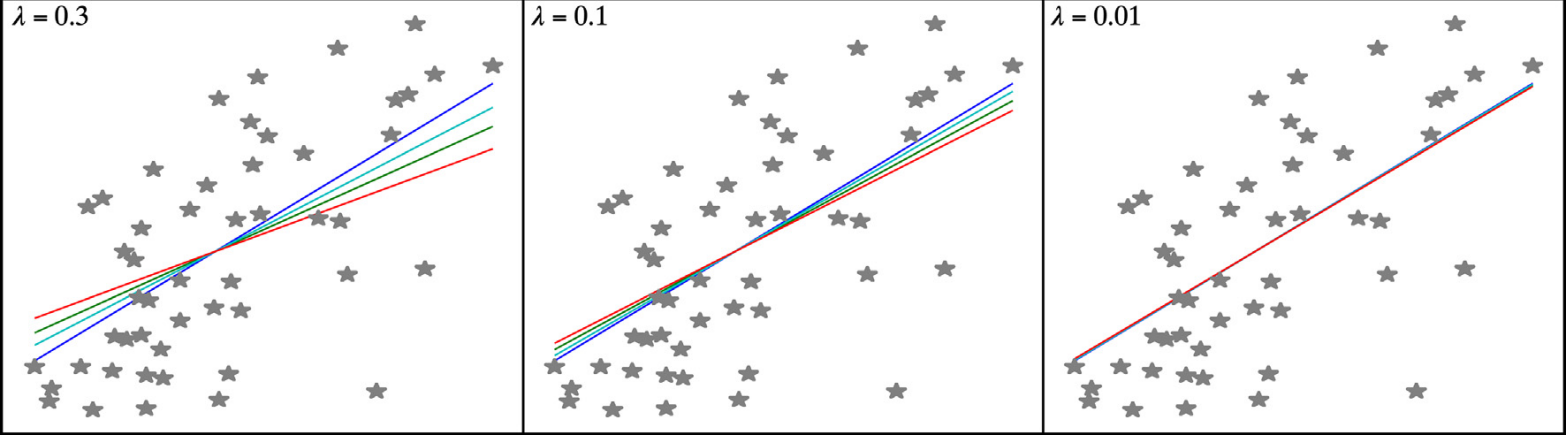
A comparison of different linear regression algorithms. The sklearn.linear_model from SciKit learn was used to generate example plots using a diabetes dataset provided in SciKit learn. Plots show that by changing the λ value, regression can be regulated such that with a small λ value, all linear regression algorithms provide similar regression. Color code: linear regression – blue, ridge regression – green, lasso – cyan, and elastic net – red. (For interpretation of the references to color in this figure legend, the reader is referred to the web version of this article.)

Linear regression algorithms are simple and constitute the most popular ML algorithms, with a wide range of applications. The standard algorithm, least squares regression, uses the sum of squared residuals as the cost function to be minimized. Least squares regression works with a simple dataset; however, with increasing complexity, the algorithm shows overfitting (low bias but large variance). To resolve this problem, several algorithms, such as the ridge model, lasso model, and elastic net, have been proposed. The cost functions in these models have been modified to increase the bias and reduce the variance. In a ridge model, a so-called L2 regularization, which is the squared value of the slope multiplied by λ, has been added to the least squares cost function. The least absolute shrinkage and selection operator (lasso) regularization (known as L1 regularization) is similar to the ridge regularization, but in this case, the added value is the absolute value of the slope multiplied by λ. The elastic net algorithm adds contributions from both L1 and L2 regularization; the cost function = min (sum of the squared residuals + λ * squared value of slope + λ * absolute value of slope). The λ parameter is a positive number that represents regularization strength. A larger λ value specifies stronger regularization, while a near-zero value removes the regularization so that all three algorithms become similar to the least squares model (Fig. 3). By changing the value of λ, it is possible to select meaningful features. Therefore, these methods can be applied to feature selection as well as to classification and regression problems [24], [28].

### 3.2 Nonlinear regression

**Fig. 4:**
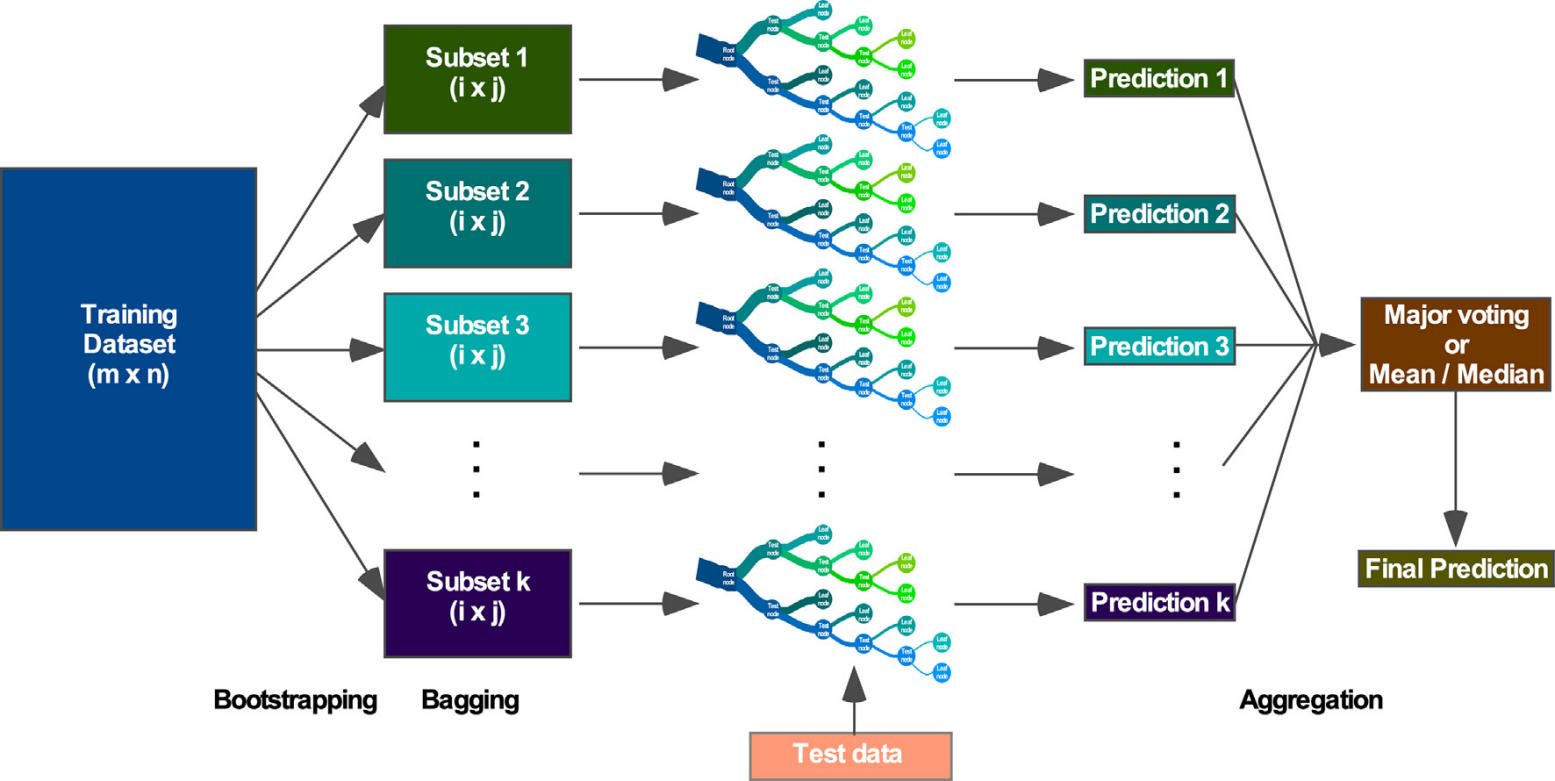
Schematic representation of random forest algorithm. The three major steps in the random forest algorithm are bootstrapping, bagging, and aggregation. During bootstrapping, the training dataset is resampled into several small datasets, which are then bagged for the decision tree. The size of the bagged dataset remains the same but bootstrapped decision trees are different from each other. All decision trees make predictions on test data, and in the aggregation step, all predictions are combined for the final prediction. For a classification problem, the final prediction is made by major voting, but for a regression problem, the final prediction uses the mean or median value.

Among the various supervised learning algorithms, the decision tree is a relatively popular predictive modeling algorithm used to classify simple data. A decision tree takes data in the root node and, according to a test rule (representing the branch), keeps growing until it reaches a decision (representing a leaf node). The internal nodes represent different attributes (features) [67]. Each internal node breaks the data into a small subset until it meets a particular condition. It is a white-box-type algorithm, as each step can be understood, interpreted, and visualized. Although the decision tree is useful for simple classification, with a larger dataset that has many features, it displays poor prediction powers due to overfitting. To resolve this problem, several advanced decision-tree-based models have been developed. The random forest algorithm randomly splits (bootstrapping) training data into several subsets (bagging) and uses each subset to build decision trees (Fig. 4). The use of multiple random decision trees for prediction increases the prediction accuracy [68]. Apart from the parallel use of random multiple decision trees, boosting algorithms, such as adaptive boosting (AdaBoost) and gradient boosting, use decision trees sequentially [69], [70]. AdaBoost usually uses one-node decision trees (decision stump), while gradient boosting uses decision trees of between 8 and 32 terminal nodes. Both adaptive and gradient boosting algorithms display better prediction performance than single decision trees. Furthermore, a more regularized gradient boosting algorithm, extreme gradient boosting (XGBoost), outperforms the former gradient boosting algorithms [71].

### 3.3 Kernel functions

**Fig. 5:**
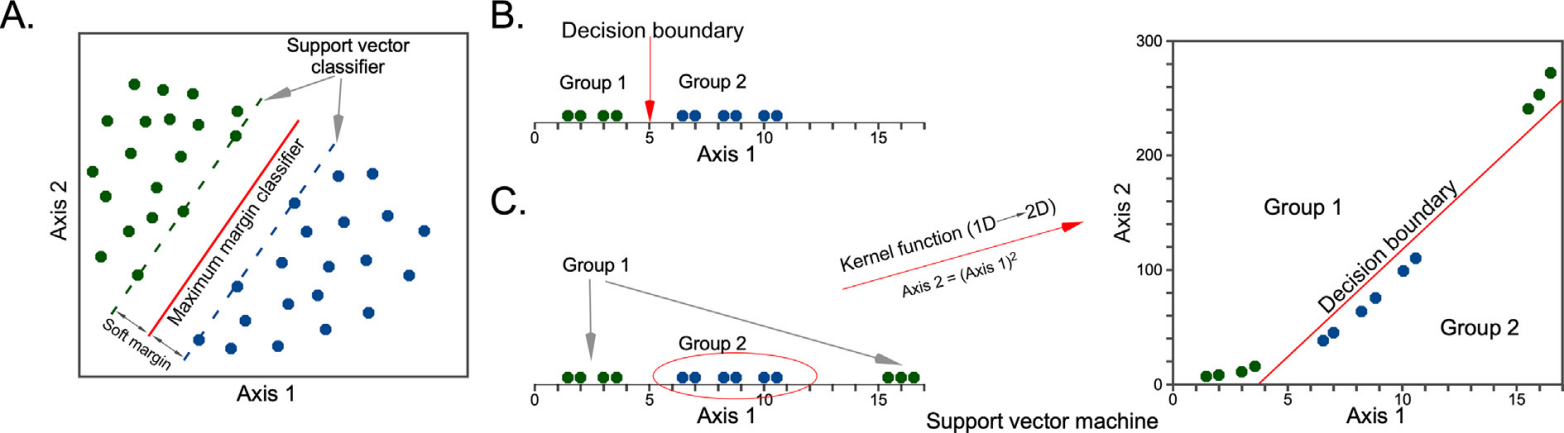
Support vector machine. (A) In a two-dimensional SVM classification system, the maximum margin classifier is a straight line (red line). Support vectors are the nearest data points from the maximum margin classifier. The distance between support vectors and the maximum margin classifier is denoted as the soft margin. (B) In a two-group, one-dimensional data space, the decision boundary is a point, as shown by the red line. (C) In a two-group one-dimensional data space where the decision boundary cannot be drawn by a point, data are transformed by a kernel function to increase the dimension. (For interpretation of the references to color in this figure legend, the reader is referred to the web version of this article.)

Kernel functions are widely used to transform data to a higher-dimensional similarity space. Kernel functions can be linear, nonlinear, sigmoid, radial, polynomial, etc. Support vector machines (SVMs) are among the most popular kernel-based algorithms that can be used not only for supervised classification and regression problems but also for unsupervised learning. In a two-dimensional space, a linear SVM classifier is defined by a straight line as a decision boundary (maximum margin classifier) with a soft margin (Fig. 5A). In this case, the soft margins are also straight lines that represent the minimal distance of any training point to the decision boundary [72]. With simple one-dimensional data, the decision boundary can be a point (Fig. 5B); however, for complex problems, the data may need to be transformed to a higher dimension to draw a decision boundary (Fig. 5C).

### 3.4 Deep learning

**Fig. 6:**
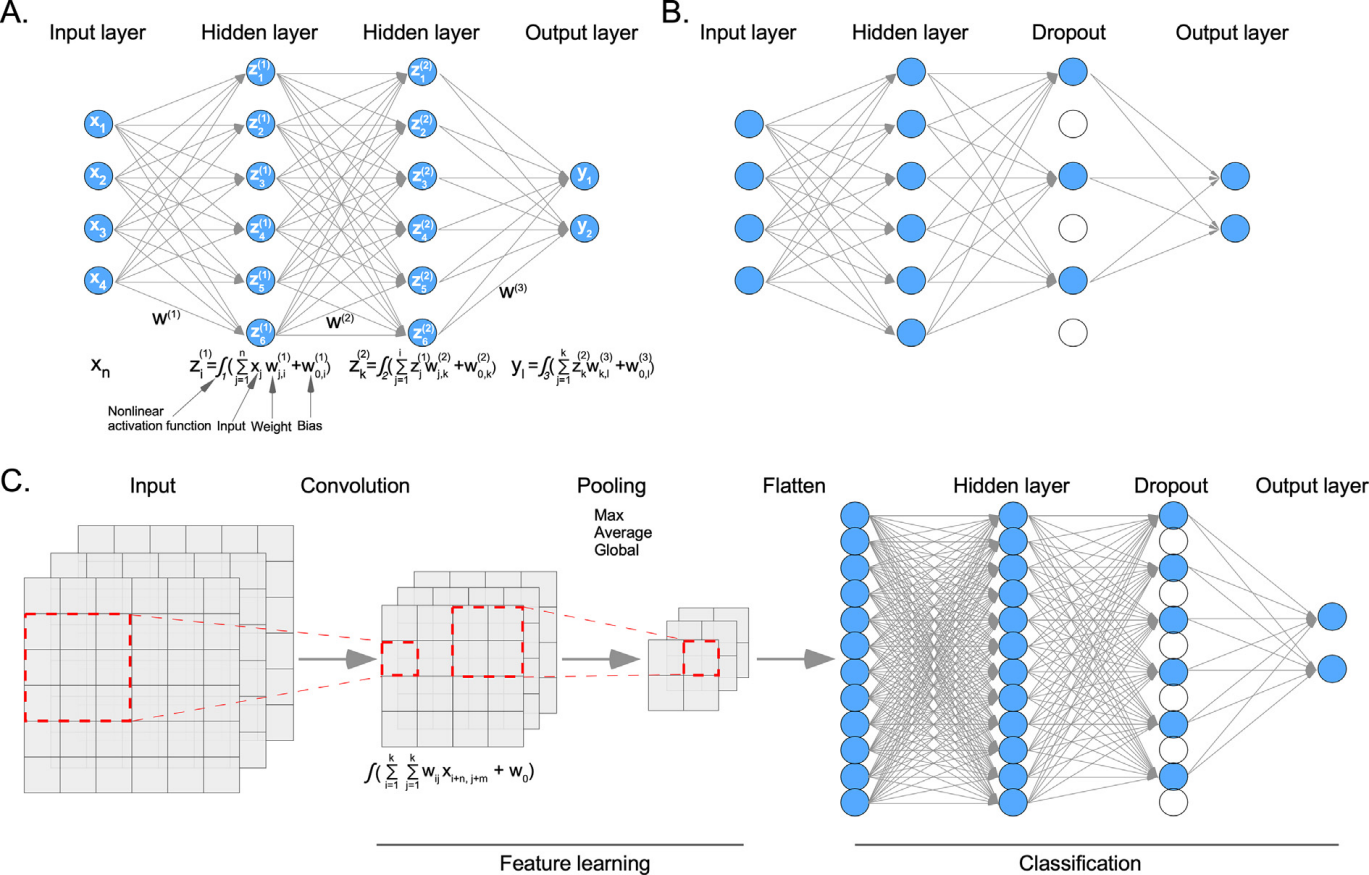
Deep learning (DL). (A) In a deep neural network (DNN) model, each node of the input data layer is fully connected to the hidden layer nodes. The first hidden layer takes input data, multiplies it by weight, and adds a bias before applying a nonlinear activation function. The second hidden layer takes the first hidden layer as input and so on until it reaches the output layer. (B) In a dropout layer, some nodes are randomly removed. (C) During the convolution, the dimension of input data is reduced using a certain kernel size (in this example, 3x3) and the activation function. Then, features are pulled for further reduction. Finally, pulled features are flattened and applied to a DNN.

DL methods are a type of ML method that can automatically discover appropriate representations for regression or classification problems upon being fed with suitable data. The model can learn complex functions and amplify important aspects to suppress irrelevant variations. During training, the algorithm takes the raw input and processes it through hidden layers using nonlinear activation functions. The algorithm tries to minimize certain cost functions by defining values for the weights and biases (Fig. 6A). Usually, gradient descent is used to find the minima. Gradients for all modules can be determined by using the chain rule for derivatives, a procedure that is known as backpropagation (starting from the output and moving toward the input) [73]. DL algorithms have been successfully employed in various domains, including image classification, because of the availability of more data than features. The development of DL models using genomic or transcriptomic data is challenging due to the limited number of samples and the presence of many features. The selection of appropriate features can reduce the feature-to-sample ratio and, thereby, prevent overfitting. Furthermore, the addition of random dropout layers can help the model learn important features and reduce overfitting (Fig. 6B).

Convolutional neural networks (CNNs) are useful for feature learning (Fig. 6C). During the convolution and pooling steps, the algorithm of a CNN learns important features [73]. CNNs are widely used for structured data, such as images; however, if the data are stored in other types of architectures, such as graphs (an example includes small-molecule drugs with multiple atoms and chemical bonds), conventional CNNs cannot be used. In this case, a different type of convolutional neural network, referred to as the graph convolutional networks (GCNs), could be applied to the graph data [74]. GCNs have especially been used to extract atomic features from drug structure (graph) data [75].

## 4 Monotherapy response prediction

**Fig. 7:**
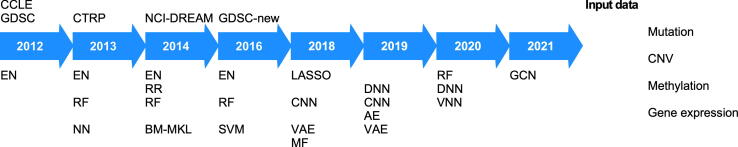
ML algorithms used in the last decade to build monotherapy response prediction. Earlier prediction models were likely developed mainly using classical ML algorithms. Later, the DL algorithms were used mostly to develop the models. The majority of the studies used multi-omics data (mutation, CNV, methylation, and gene expression) collected from large screening studies such as CCLE, GDSC, CTRP, etc. EN – elastic net, RF – random forest, NN – neural network, RR – ridge regression, BM-MKL – Bayesian multitask multi-kernel learning, SVM – support vector machine, LASSO - least absolute shrinkage and selection operator, CNN – convolutional neural network, DNN – deep neural network, AE – autoencoder, VAE – variational autoencoder, MF – matrix factorization, VNN – visual neural network, GCN – graph convolutional network.

Currently, only a few drug response prediction tools are available for clinical use. In fact, a couple of linear regression prediction models are currently being used for certain types of cancers. A supervised classification model using a 70-gene signature was developed in 2002 to predict chemotherapy responses in breast cancer [76]. The method was patented as MammaPrint and is currently used in the clinic for patients with early-stage breast cancer. Later, a similar method was developed in which a linear regression model based on the scores of a 21-gene signature (Oncotype DX) was used to predict the chemotherapy responses in early-stage, estrogen-receptor-positive, HER2-negative invasive breast cancer [77]. Furthermore, a 50-gene signature was employed in multivariate supervised learning (PAM50 or Prosigna, a breast cancer prognostic gene signature assay) to predict treatment responses in breast cancer [78]. Aside from these simple, cancer-subtype-specific prediction models that are currently available in the clinic, most other studies regarding monotherapy predictions are still in the preclinical phase. Fig. 7 shows an overview of the methods that have been used to develop monotherapy prediction models in the past decade (a brief overview is included in Table 1).

**Table 1:** Studies predicting monotherapy responses.

Year	Data	Features	Algorithm	Ref.
2012	GDSC	Mutation, CNV, gene expression	Elastic net	[28]
	CCLE	Mutation, CNV, gene expression	Elastic net	[24]
2013	CCLE, GDSC	Gene expression (1000 selected genes)	Elastic net and other	[54]
	CTRP	Mutation, CNV	Elastic net	[30]
	GDSC	Selected genomic features	Neural networks and random forests	[80]
2014	GDSC, clinical data	Gene expression	Ridge regression	[79]
	CCLE, GDSC	Mutation, CNV, gene expression	Elastic net and ridge regression	[50]
	GDSC, CCLE, NCI	Gene expression (1000 selected genes)	Random forest	[55]
	NCI-DREAM	Mutation, CNV, gene expression, proteomic	BM-MKL	[49]
2015	GDSC, CCLE	Gene expression	Cell line-drug network model	[82]
2016	NCI	Mutation, CNV, gene expression, RPLA, miRNA	Random forest and support vector machine	[81]
	GDSC 2	Mutation, CNV, gene expression, methylation	Elastic net and random forest	[27]
	LINCS	Gene expression	DNN	[88]
2018	AML patient and cell line data	Gene expression	VAE + LASSO (DeepProfile)	[99]
	GDSC	Genomic fingerprints	CNN	[91]
	AML patient and cell line data	Gene expression, mutation, CNV, methylation	Network-based gene-drug associations	[87]
	PharmacoDB, CMap	Gene expression	VAE (Dr.VAE)	[59]
	CCLE, GDSC	Gene expression	Recommender systems	[94]
2019	GDSC	Gene expression	DNN	[90]
	TCGA, CCLE	Mutation, gene expression	VAE, DL (DeepDR)	[60]
	GDSC	Mutations and CNV	CNN ((tCNNS)	[105]
	GDSC	Mutation, CNV, gene expression.	DL (MOLI)	[92]
	GDSC, CCLE	Gene expression	Autoencoder (DeepDSC)	[61]
2020	PDXGEM	Gene expression	Random forest	[106]
	GDSC, KEGG, STITCH	Gene expression, pathway	DL	[89]
	GDSC, CCLE, CTRP	Gene expression, mutation, CNV, methylation	VNN	[62]
	van de Wetering et al. [108], Lee et al. [109]	Gene expression, pathway	Ridge regression	[107]
2021	GDSC	Mutations and CNV	Graph convolutional network	[104]

### 4.1 Classical ML models in monotherapy prediction

Sparse linear regression models have been used to predict drug sensitivity in initial large-scale pharmacogenomic studies with cell lines from various cancers [24], [28], [30]. These studies combined genomic features with transcriptomic features from cell lines and correlated them with corresponding drug sensitivity scores. The ridge regression and elastic net algorithms were predominantly employed for predictions [24], [28], [30], [50], [79]. However, due to the linear nature of the algorithms and the use of many features, these models could easily become overfitted.

As discussed above, the performance of prediction algorithms is largely influenced by biological feature selection [54], [55], [80], [81]. Prediction performance can further be improved by incorporating information on the similarity between cell lines and drugs [82]. Cell lines with a similar gene expression profile show similar responses to a specific drug, while drugs with a similar chemical structure display similar inhibitory effects toward different cell lines. Therefore, a dual-layer network model that also considers similarity information outperforms linear models [82]. Likewise, a method based on a heterogeneous network in which the relationships among drugs, drug targets, and cell lines were explicitly incorporated was shown to better capture the relationship between cell lines and drugs [83]. Collectively, a predictive model with selected features performs better, and the addition of network features improves the prediction accuracy.

The community-based NCI-DREAM study used a limited number of samples with a large number of genomic, transcriptomic, and proteomic features [49]. The NCI-DREAM initiative developed 44 different drug sensitivity prediction models, with the Bayesian multitask multikernel learning (BM-MKL) models performing relatively better than other models. BM-MKL includes Bayesian inference, multitask learning, multiview learning (multiple data view), and kernelized regression [49], [84], [85]. The standard model, kernelized regression, is a nonlinear classification algorithm similar to SVMs. Unlike the elastic net, kernelized regression captures the nonlinear relationship between drug sensitivity and genomic or transcriptomic features but simplifies the process by using a single component for the predictions.

Besides using genomic or transcriptomic features to predict drug sensitivity, the chemical and structural properties (also known as descriptors) of drugs have been incorporated into the learning algorithms. Combining drug descriptors with genomic or transcriptomic data allows for the simultaneous prediction of multiple drug responses from a single model, although it is a challenging task due to the further increase in the total number of features [86]. Likewise, in a study with multicancer and multidrug associations, a disease-specific multi-omics approach to predicting gene-drug association was adopted in which each gene was checked for a pathway association [87]. The method is useful for identifying critical regulatory genes that can be targeted by a drug.

### 4.2 Deep neural networks in monotherapy prediction

Although DL has long been widely used in several areas of medical science and drug discovery platforms, it has recently been applied to drug response prediction as well. Initially, feedforward deep neural networks (DNNs) were applied to develop models using selected genomic features [80] or transcriptomic data [88]. Later studies incorporated selected gene expression features with pathway information to build DNN models [89], [90]. In any case, all these DNN models have been shown to outperform classical ML models.

A CNN was used in the Cancer Drug Response Profile scan (CDRscan) study, in which convolutions were applied separately to genomic fingerprints of cell lines and molecular fingerprints of drugs [91]. After convolution, those two sets of features were merged and used with the drug response data to develop a DNN model. Because a CNN learns important features during training [73], the CDRscan method displays considerably higher robustness and generalizability. A similar model (MOLI) was developed using somatic mutations, CNVs, and gene expression data from GDSC [92]; the model was later validated with PDXs and patient samples.

### 4.3 Matrix factorization and factorization machines in monotherapy prediction

**Fig. 8:**
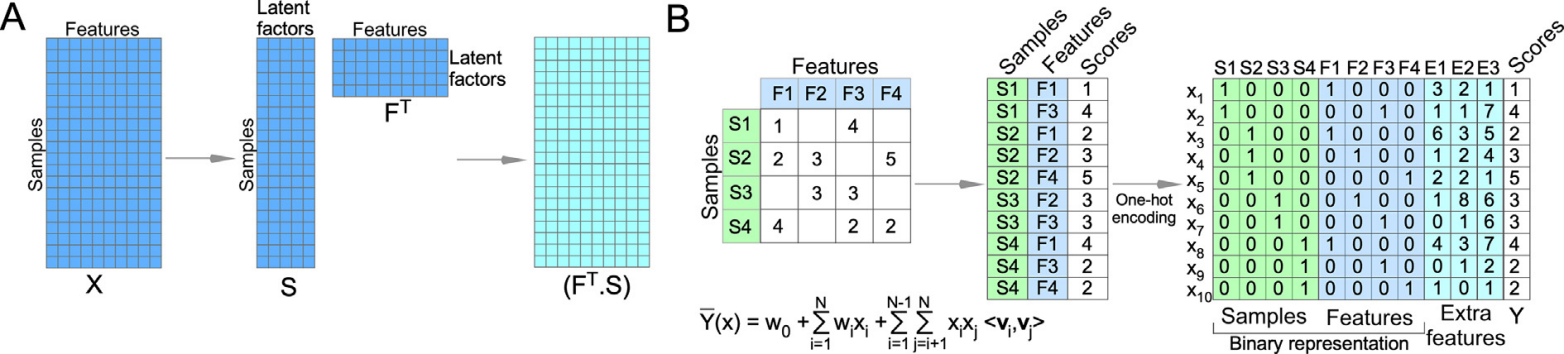
Matrix factorization and factorization machine. (A) In MF, a matrix is decomposed into two lower-dimensional matrices with the same latent factor. The dot product of lower-dimensional matrices is used to reconstitute the new matrix to calculate the loss function. (B) An FM transforms sample and features data to the binary representation and can incorporate additional features.

Matrix factorization (MF) is a supervised learning method that has been widely used in popular e-commerce ML recommender systems [93]. MF takes high-dimensional data, with missing information, as input and decomposes it into lower-dimensional matrices with the same numbers of latent factors (Fig. 8A). The learning algorithms in recommender systems are not general and must be tailored to each specific model. A modified recommender system was developed (CaDRReS) in which cell line features were first calculated using gene expression information [94]. The MF method determined the pharmacogenomic space (the dot product of the cell line vector and the drug vector), and drug sensitivity was computed using a specific linear algorithm. The model was compared to other ML algorithms and was found to perform similarly to the elastic net. Because the model provides a projection of cell lines and drugs into the pharmacogenomic space, it is easy to explore relationships between drugs and cell lines [94].

In a recommender system, MF cannot add additional features and cannot predict a completely new item, as the method is highly dependent on data from input features. To resolve those issues, in 2010 Rendle introduced a generalized algorithm, the factorization machine (FM)) [95]. FMs are SVM-like predictors but can handle data with high sparsity (Fig. 8B). Classical FMs can easily handle second-order feature combinations but struggle with higher-order feature combinations. Blondel et al. proposed an updated algorithm for the easy handling of higher-order feature combinations, referred to as higher-order factorization machines (HOFMs) [96]. So far, HOFMs have not been used in monotherapy response prediction; however, they have been employed to predict drug combinations (as described below).

### 4.4 Autoencoders in monotherapy prediction

**Fig. 9:**
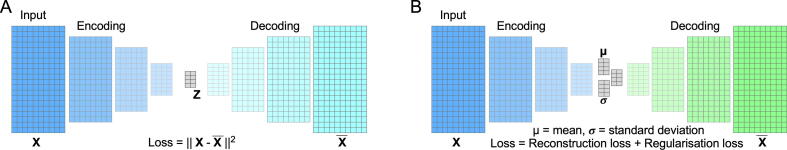
Autoencoder and variational autoencoder. (A) The autoencoder determines latent variables by reducing the dimensions during encoding. Then it decodes the data into a similar form using the latent variables. (B) VAE uses a similar process unless the latent variables are replaced by the mean and standard deviation.

An autoencoder is an unsupervised DL model that can be used to reduce the dimension of features. An autoencoder learns hidden (latent) variables from the observed data through the mapping of higher-dimensional data onto a lower-dimensional latent space. An autoencoder consists of two different types of layers: encoding layers and decoding layers, with encoding layers projecting higher-dimensional input data onto lower dimensions and decoding layers reconstructing the lower-dimensional data back to the higher-dimensional data similar to input (Fig. 9A). The loss function is the least squares difference between the input and output vectors. In this case, if the decoding weights correspond to the encoding weights, the output will be the same as the input (deterministic encoding). In general, an autoencoder uses nonlinear activation functions for data compression and can discover nonlinear explanatory features; therefore, it can be used to reduce gene expression features and uncover a biologically relevant latent space [61], [97].

Besides the traditional autoencoder, the variational autoencoder (VAE) replaces the deterministic bottleneck layer with stochastic sampling (mean and standard deviation) vectors (Fig. 9B). The model includes regularization losses by adding a Kullback-Leibler (KL) divergence term. This reparameterization allows for backpropagation optimization and for learning the probability distribution of each latent variable instead of directly learning the latent variables [98].

The DL model to predict drug response (DeepDR) combined mutational data with gene expression data to develop a monotherapy prediction model, implementing an autoencoder for both mutational and gene expression data [60]. In this model, the autoencoder was first applied to the TCGA data to transform the mutational and gene expression features into a lower-dimensional representation. The encoded representations of the TCGA data were linked to a feedforward neural network trained on CCLE data for monotherapy prediction. The use of autoencoding increased the sample number in the prediction model and, therefore, displayed better prediction performance. Besides an autoencoder, a VAE was used to reduce the higher-dimensional acute myeloid leukemia (AML) patient gene expression data to an 8-dimensional representation, and the VAE was then used to build a linear regression model (lasso) for drug response prediction [99]. Later, a drug response VAE (Dr.VAE) was developed using drug-induced gene expression perturbation [59]. This study used a semi-supervised VAE to predict monotherapy responses using cell line data, and the model was shown to perform better than several linear or nonlinear algorithms. The use of drug-induced gene expression perturbation seems to be useful in determining pathways that regulate drug response and therapy resistance [100]. Nevertheless, anomaly detection with density estimation can improve the prediction accuracy through false positive detection, but this still needs to be implemented [101].

### 4.5 Graph convolutional networks in monotherapy prediction

Therapy response prediction using multiple drugs requires the incorporation of chemical information about the drugs. This can be done in several ways. The 2D molecular fingerprint (also known as the Morgan fingerprint or circular fingerprint) is commonly measured by the extended-connectivity fingerprint (ECFP) algorithm [102]. This algorithm determines partial structures and converts them into a binary representation. Similarly, the 3D fingerprint descriptor collects 3D information, including electrostatics and molecular shape. The simplified molecular input line entry specification (SMILES) representation was developed by Weininger and provides a linear notation method [103]. SMILES can be used directly by a CNN. Molecular graphs are another type of flexible representation of small-molecule drugs. The GraphDRP study used a molecular graph representation in a GCN to extract molecular features from drugs [104]. At the same time, a CNN was used to extract genomic features from cell lines. Then, the features from the GCN and CNN were combined and fed into the fully connected feedforward neural network for drug sensitivity prediction. The GCN model was compared to a recently developed CNN model using the SMILES format to describe the drugs and was found to perform better, suggesting that the use of graph data for drugs improves predictive performance [105].

### 4.6 Visible neural networks in monotherapy prediction

Model interpretation is an important research area in ML that seeks to explain the model’s internal rationality of a prediction. Biological ML models that were developed with prior knowledge of network or structural data can be explained relatively easily. A so-called visible neural network (VNN) incorporates genomic or transcriptomic data considering the cellular architecture and signaling pathways [62]. Chemical information about drugs was separately processed and then combined with the embedding genotype data to develop the final prediction model (DrugCell). The DrugCell method was compared to the elastic net and other DNN models and found to have a similar or better predictive performance.

### 4.7 PDXs and organoids in monotherapy prediction

Although most studies used cell line data to develop ML models, recently the PDXGEM study applied PDXs to develop an ML model [106]. In this study, drug activity was calculated as a percentage of tumor volume changes. Baseline gene expression profiling data were used to develop the model. Another recent study used data from 3D organoid culture models and applied protein–protein interaction networks [107]. The model was trained with pharmacogenomic data from two previous studies using ridge regression [108], [109]. This study developed a clinically relevant prediction model that was also useful in identifying predictive biomarkers [107]. Collectively, the use of PDXs and organoids in model development increases the probability of successful clinical applications.

## 5 Drug synergy prediction

The use of monotherapy in cancer treatment is relatively rare, and most cancer patients are treated with a combination of several drugs. Cancer cells can easily develop resistance to monotherapy, while the development of resistance to several drugs can be difficult or take longer. Therefore, combinatorial therapies are preferred over monotherapy in clinics for cancer treatment. A combination of multiple drugs can have three different effects: additive, antagonistic, and synergistic. The additive effect can be considered a neutral effect, while the antagonistic effect is negative. The synergistic effect is preferable. Thus, predicting drug synergy will be highly beneficial for selecting effective combinations for cancer treatment.

Drug synergy is usually calculated by a cell viability matrix, in which a wide range of single and combinatorial drug effects are noted. The Institute for Molecular Medicine Finland (FIMM) developed an experimental-computational pipeline to measure and visualize synergy from drug combinations [110]. It allows for the simultaneous measurement of several synergy scores, such as Bliss independence [111], Loewe additivity [112], highest single agent (HSA) [113], and zero interaction potency (ZIP) [114]. Later, the study was extended to the prediction of drug combinations [115]. Combenefit is yet another program for calculating synergy scores, in particular Loewe additivity [116].

Several attempts have been made to identify drug synergy using cell lines from different cancers [117], [118], [119], [120], [121], [122], [123]. These studies provided an initial framework for developing ML algorithms for predicting drug synergy. A list of available in silico drug synergy prediction models is given in Table 2.

**Table 2:** Studies predicting drug synergy.

Year	Study name	Data	Algorithm	Ref
2015	RACS	DCDB [151], KEGG, NCI-DREAM	Semi-supervised learning	[118]
2017	Li et al.	DREAM [128]	Random forest	[130]
	Gayvert et al.	Held et al. [120]	Random forest	[140]
	SynGeNet	LINCS L1000, Held et al. [120]	Network-based	[136]
2018	Xia et al.	NCI-ALMANAC [141]	DL	[142]
	Deep Synergy	O’Neil 2016 [122]	DL	[143]
	Deep belief	DREAM [128]	Restricted Boltzmann machine	[150]
2019	SynGeNet	LINCS L1000	Network based	[137]
	DREAM	CNV, mutation, methylation, and gene expression	Multiple	[117]
	DDIGIP	DrugBank, SIDER, OFFSIDES	Regularized Least Squares	[126]
	Cuvitoglu et al.	DCDB [151], Cmap [131]	Naive Bayes, Support Vector Machines, and Random Forest	[132]
	Malyutina et al.	O’Neil 2016 [122]	Elastic net, random forest, support vector machine	[115]
2020	Deep graph	O’Neil 2016 [122]	graph convolutional network	[147]
	comboFM	NCI-ALMANAC [141]	Higher-order factorization machines	[145]
2021	CellBox	Perturbation data [134]	ODE	[133]
	AuDNNsynergy	O’Neil 2016 [122]	Autoencoder	[146]
	TranSynergy	O’Neil 2016 [122]	Transformer boosted DL	[144]

### 5.1 Drug synergy prediction using conventional ML methods

In silico methods integrating molecular data with pharmacological data could potentially identify drug combinations with some limitations [124]. A heterogeneous network-assisted inference (HNAI) framework was developed using drug-drug interaction pairs connecting approved drugs, phenotypic similarity, therapeutic similarity, chemical structure similarity, and genomic similarity using naive Bayes, decision tree, k-nearest neighbor (KNN), logistic regression, and SVM algorithms [125]. Then, the DDIGIP method, in which the Gaussian interaction profile (GIP) kernel and the regularized least squares (RLS) classifier were implemented, was based on drug-drug interactions (DDIs) [126]. DDIGIP used the similarity of drug features extracted from drug substructures, targets, transporters, enzymes, pathways, indications, side effects, offside effects, and drug-drug interaction data. Collectively, these methods give valuable insights into drug-drug interactions but cannot provide information about whether certain drug combinations will be effective for a specific patient. Gene expression data were used at a limited scale to predict the effect of drug combinations by the Petri net model [127], but the model requires gene expression profiles for every drug pair, which limits its practical applications.

In a DREAM challenge, the human diffuse large B-cell lymphoma (DLBCL) cell line OCI-LY3 was treated with 91 compound pairs of 14 drugs. The drug-induced genomic residual effect model—which combined similarity and dissimilarity in compound activity incorporating drug-induced gene perturbation, dose–response, and pathway information—was reported to outperform 30 other models [128], [129]. Although the accuracy of the predictive models was not optimal for practical applications, this study raised the probability of building computational predictive models for drug synergy prediction. The gene expression perturbation data generated in this project are valuable for other studies and can be used to train random forest models with the biological and chemical properties of drugs, such as physicochemical properties, target network distances, and targeted pathways [130]. Similarly, Cuvitoglu et al. extracted the drug perturbation set of genes for each drug from the transcriptome profile of Cmap data [131] and calculated six different features: the distance between two drugs (M1), the mutual information about biological processes (M2), the gene ontology similarity (M3), the overlap of drug perturbation sets (M4), the betweenness centrality of the drug combination network (M5), and the degree of the drug combination network (M6) [132]. Three models were developed using a naive Bayes classifier, an SVM, and a random forest algorithm. Different features were tested, and models combining the M5 and M6 features performed the best. In addition, the CellBox method used perturbation data of the melanoma SK-Mel-133 cell line treated with 12 different drugs [133], [134]. Using nonlinear ordinary differential equations (ODEs), CellBox provided an interpretable ML system that can be used to predict drug combinations in a dynamic system. This study provided mechanistic insights for designing a combination therapy with an understandable predictive model. Taken together, these studies suggest that drug perturbation data provide important information about the regulation of biological features that can be used to develop efficient ML models [100].

Models integrating the signaling network or pathway map have been used to detect drug combinations with limited general applications [135], [136], [137]. Similarly, synergy prediction models developed with naive Bayes classifiers [138] and random forest algorithms [139], [140] had limited use for specific cell models. Collectively, synergy prediction models developed using classical ML algorithms displayed acceptable predictive performance with specific datasets but largely lacked generalizability.

### 5.2 Drug synergy prediction using DL

DL has been employed in the prediction of drug synergy. Using the NCI-ALMANAC database [141], it has been demonstrated that the use of gene expression, microRNA, and proteome data, along with drug descriptors, provides the highest prediction capability with feedforward neural networks [142]. This model used two submodels to separately process drug descriptors and gene expression, microRNA, and proteome data. The submodels were fully connected neural networks that helped reduce the dimensionality of the data before they were fed into the final model. This study provided important insight into the use of DL in feature selection and model development.

The DeepSynergy study [143] used a previously published drug synergy dataset [122] to build a DL model and compared it with several classical ML methods, such as gradient boosting, random forest algorithms, SVMs, and elastic nets. This feedforward DL model, which used gene expression data with the chemical features of both drugs to predict Loewe additivity, achieved considerable accuracy. The use of DL allowed the model to perform better than other ML algorithms, but it should also be tested with unknown samples.

Recently, transformer boosted DL (TransSynergy) was developed, in which three components were used: input dimension reduction, a self-attention transformer, and a fully connected output layer [144]. The input vector contained selected features from two drugs (drug-target interaction profile) and the cell line (gene expression). A fourth dimension was added if both gene expression and gene dependency were used. The use of cell-line-gene dependency, gene-gene interaction, and drug-target interaction provided TransSynergy with a considerably higher predictive performance and allowed the cellular effect of drug actions to be explained. These methods provided a significant improvement over traditional ML mechanisms due to appropriate feature learning. However, all those models used cell line synergy data [122], which might limit their application in preclinical and/or clinical trial settings.

### 5.3 Synergy prediction with a higher-order factorization machine

An HOFM model [96] was used in comboFM to capture fifth-order feature combinations using data from two drugs, cell lines, and dose–response matrices [145]. The model integrated chemical descriptors of drugs and gene expression data of cell lines as additional features. comboFM was trained with a part of the NCI-ALMANAC data, while the other part of the data was used for predictive performance testing. The fifth-order comboFM was found to perform significantly better than second- and first-order predictors, suggesting that the use of higher-order feature combinations can improve predictive performance.

### 5.4 Synergy prediction using an autoencoder

An autoencoder has also been employed to predict drug synergy [146]. AuDNNsynergy used multi-omics data from CCLE and TCGA databases combined with previously published drug synergy data [122]. In this study, three independent autoencoders were used to reduce the dimensions of TCGA gene expression, mutation, and copy number data. The reduced dimensions were then combined with drug combination data to develop the model. The model was compared with the recently developed DeepSynergy model and was shown to perform better [143], suggesting that feature reduction using an autoencoder and the use of multi-omics data influence predictive performance.

### 5.5 Synergy prediction with a graph convolutional network

A graph convolutional network (GCN) model was described (DeepGraph) in which a drug-drug synergy network, a drug-target interaction network, and a protein–protein interaction network were used to build a cell-line-specific model [147]. In the DeepGraph study, a cell-line-specific multirelational network graph was generated and fed into the GCN encoder. A four-layer neural network with a relu activation function was used for encoding, and a sigmoid activation function was used for the embedding output vector. The matrix decoder was used to decode the embedding vector, which predicts the synergy score [74]. The prediction performance of DeepGraph was comparable to that of DeepSynergy. Because the DeepGraph method used a cell-line-specific drug-protein network and protein–protein interaction network and because only limited data for drug-protein interactions were available, the method’s performance might be biased.

### 5.6 Restricted Boltzmann machine for predicting drug synergy

The restricted Boltzmann machine (RBM) is a generative probabilistic model that has been widely used for handling higher-dimensional data [148]. The RBM is similar in function to an autoencoder and can be used to extract meaningful features from higher-dimensional data. Furthermore, multiple RBMs can be stacked to form a deep belief network, which allows unsupervised and supervised data to be combined. RBMs have been used to identify gene expression biomarkers that can help predict clinical outcomes [149]. Chen et al. used RBMs to develop a deep belief network [150] from the DREAM consortium’s drug target information and baseline gene expression data [128]. Although the model was compared with existing DREAM consortium models and was shown to outperform these models, the leave-one-out approach that was adopted in this study was not comparable to the original DREAM consortium models, which were compared with external data.

## 6 Limitations in the development of clinically relevant predictive models

Currently, most ML models have been developed using cell line data. Cell line data are robust, relatively easy to generate, and useful for hypothesis generation. However, cell line data must be complemented with more disease-relevant patient data. A large-scale pharmacogenomic study using patient data is currently technically difficult because it requires a lot of primary patient materials. This can potentially be overcome by using PDXs. The recent development of PDX repositories will support large-scale clinically relevant studies in the near future [37], [38], [39], [40].

Most tumors grow in a multicellular environment in which the surrounding cells create a favorable microenvironment for tumor growth. Prediction models based on cell line data do not capture the microenvironment’s contributions and might therefore never reach the level of accuracy that is necessary in the clinic. Cultured tumor organoids can likely mimic the microenvironment of a patient’s tumor [107]. However, currently, only limited pharmacogenomic data from tumor organoids are available.

Several recent models used multi-omics data to build predictive models [62], [87], [92]. Although the use of multi-omics data can improve the prediction performance and can be very useful for research purposes, it limits the practical use of the models in the clinic. For prediction purposes, it would be costly and time-consuming to determine mutations, CNVs, promotor methylation, protein expression, gene expression, etc. for each patient separately. Gene expression data can potentially reflect most cellular processes because mutations, CNVs, and promotor methylation might ultimately determine gene expression changes.

Most gene expression data currently available involve the baseline expression of genes and do not reflect drug-induced perturbations [24], [28], [30], [80]. A few studies provided a limited number of drug-induced perturbation data, which were found to be very useful for feature selection [59], [134]. Thus, large-scale drug-induced perturbation studies will help to develop better predictive models.

Nevertheless, drug synergy prediction is an important concept that will have numerous uses in the clinic. At the same time, a combination of several drugs can have severe adverse effects. Thus, a comprehensive method is needed that will not only determine drug synergy but also incorporate the adverse effect of drug combinations. Knowledge of safe and unsafe combinations of drugs was used to build a linear regression prediction model [152], [153], [154]. However, the model did not incorporate any biological data to elucidate patient-specific side effects.

Several studies have highlighted implementation challenges encountered in precision medicine solutions [155], [156]. These challenges include data preprocessing, unstructured clinical text processing, medical data processing and storage, and environmental data collections. Apart from these challenges, the major challenge might be the redesigning of clinical decision support systems so that they can incorporate molecular, omics, and environmental aspects of precision medicine. A comprehensive support system is desirable to facilitate the curation of data from different sources and multiple scales and to promote the interaction between bioinformatics and clinical informatics [155]. Building such a system requires solving many integration and standardization issues.

As pointed out by many studies, model explainability, high-quality training data, and collaborations between medical experts and computational experts are some of the key factors affecting the success of ML solutions for drug response prediction in cancer treatment [9], [157]. Although much omics information is available and many theoretical frameworks exist, hands-on ML tools targeted at physicians and medical professionals are scarce. In that regard, various cloud-based cancer prediction tools, such as OASISPRO [158], can be introduced to make ML solutions suitable for massive clinical practice. The study gave an overview of general-purpose multi-omics tools that can be useful for gene identification and cancer subtyping [159].

Clinical trials are essential for clinical research in general and cancer treatment in particular. The three-phase trial approach is considered standard practice but is designed primarily for gradually improving treatments. Our ability to understand and treat cancer has, however, evolved over time [21]. Because of the immense role of ML in both clinical trials and clinical practice, the inclusion of ML in regulatory frameworks is unavoidable.

## 7 Conclusion

The development of predictive models for monotherapy and combinatorial therapies is important but highly challenging. The recent advancement in ML algorithms holds promise for the development of clinically relevant predictive models. Furthermore, more pharmacogenomic data from disease-relevant organoids and PDXs are becoming available, allowing clinical biases to be overcome. Massive computational power is within easy reach for handling a large amount of data that is exponentially increasing. In the near future, the current lack of clinically relevant pharmacogenomic data might also be overcome. Therefore, although current predictive models are far from being ready for clinical use, they show us a clear path toward precision medicine.

## CRediT authorship contribution statement

**Raihan Rafique:** Writing - original draft, Writing - review & editing. **S.M. Riazul Islam:** Writing - original draft, Writing - review & editing. **Julhash U. Kazi:** Conceptualization, Writing - original draft, Writing - review & editing.

## Declaration of Competing Interest

The authors declare that they have no known competing financial interests or personal relationships that could have appeared to influence the work reported in this paper.
